# Imaging Findings of Atypical Spindle Cell/Pleomorphic Lipomatous Tumors: A Case Series and Systematic Review

**DOI:** 10.7759/cureus.94254

**Published:** 2025-10-09

**Authors:** Masaya Kawaguchi, Hiroki Kato, Akihito Nagano, Natsuko Suzui, Tatsuhiko Miyazaki, Shingo Omata, Yoshifumi Noda, Abdelazim E Elhelaly, Hirohiko Imai, Masayuki Matsuo

**Affiliations:** 1 Department of Radiology, Gifu University, Gifu, JPN; 2 Department of Orthopaedic Surgery, Gifu University Hospital, Gifu, JPN; 3 Department of Pathology, Gifu University Hospital, Gifu, JPN; 4 Department of Frontier Science for Imaging, Gifu University, Gifu, JPN; 5 Department of Food Hygiene and Control, Faculty of Veterinary Medicine, Suez Canal University, Ismailia, EGY; 6 Innovation Research Center for Quantum Medicine, Graduate School of Medicine, Gifu University, Gifu, JPN; 7 Center for One Medicine Innovative Translational Research (COMIT), Gifu University, Gifu, JPN

**Keywords:** atypical spindle cell/pleomorphic lipomatous tumor (ascplt), ct, mri, soft-tissue tumor, systematic review

## Abstract

This study aimed to examine the CT and MRI features of atypical spindle cell/pleomorphic lipomatous tumors (ASCPLTs) through a case series and systematic review.

A literature search was conducted using MEDLINE and Scopus databases, covering the period from January 1, 2017, to June 9, 2025. The following keywords were used: (“atypical spindle cell lipomatous tumor”) OR (“atypical pleomorphic lipomatous tumor”) OR (“atypical spindle cell/pleomorphic lipomatous tumor”). The study included five patients from our institution with histopathologically and immunohistochemically confirmed ASCPLTs, along with 18 additional cases identified from 18 published articles. Imaging findings were reviewed retrospectively. A total of 23 patients with ASCPLTs were analyzed (14 male patients and nine female patients; median age, 60 years; age range, 13-87 years). The median lesion size was 115 mm. Fourteen lesions (61%) were superficial, and nine (39%) were deep. Tumor margins appeared well-defined in 20 (87%) cases and ill-defined in three (13%) cases. A capsule was identified on T2-weighted images in 9/12 (75%) cases. Fatty and non-fatty components were observed in 14/18 (78%) and 15/18 (83%) cases, respectively, with non-fatty components predominating in 13/18 (72%) cases. Imaging patterns were classified as follows: atypical lipomatous tumors in 3/17 (18%) cases, spindle cell lipoma in 3/17 (18%), myxoid liposarcoma in 5/17 (30%), dedifferentiated liposarcoma in 4/17 (22%), and undifferentiated pleomorphic sarcoma in 2/17 (12%).

Key imaging characteristics of ASCPLTs included large size (>100 mm), superficial location, well-defined margins, presence of a capsule, and predominance of non-fatty components. While ASCPLTs frequently contained fat, their imaging presentations varied widely.

## Introduction

Atypical spindle cell/pleomorphic lipomatous tumors (ASCPLTs) are benign adipocytic tumors characterized by a variable mix of mildly to moderately atypical spindle cells, adipocytes, lipoblasts, pleomorphic cells, multinucleated giant cells, and either a myxoid or collagen-rich extracellular matrix. ASCPLTs primarily affect middle-aged individuals, with peak incidence occurring in the sixth decade of life and a male-to-female ratio of 3:2 [1-4]. The median tumor size is 5 cm, ranging from 0.5 to 28 cm [4]. Around two-thirds of these tumors are located in the limbs and limb girdles, especially in the hands and feet, with approximately equal occurrence in both superficial and deep layers [1-4]. ASCPLTs are characterized by a low rate of local recurrence (10%-15%) following incomplete excision, no evidence of distant metastasis, and rare sarcomatous transformation [1,2,5]. Complete surgical removal typically results in an excellent prognosis [1]. Therefore, accurate imaging diagnosis of ASCPLTs may help avoid overtreatment.

The primary pathological differential diagnoses of ASCPLTs include a spectrum of adipocytic tumors, benign, intermediate, and malignant, such as spindle cell lipoma, atypical lipomatous tumor, myxoid liposarcoma, and dedifferentiated liposarcoma [2]. Similar to pathological findings, radiological differentiation between ASCPLTs and other fatty tumors, including sarcomas, is quite challenging due to overlapping imaging features [2]. While numerous case reports with radiological images have been published [5-22], detailed descriptions of the imaging features of ASCPLTs are limited in the literature [2]. Therefore, the purpose of this study was to identify the distinctive imaging features of ASCPLTs and to provide insights for differentiating ASCPLTs from other soft-tissue tumors through a case series and systematic review.

## Case presentation

This study received approval from the Human Research Committee of our hospital’s institutional review board, which also waived the requirement for written informed consent. The study was conducted in compliance with the Health Insurance Portability and Accountability Act of 1996. We identified patients at our institution with ASCPLTs confirmed through both histopathological and immunohistochemical analysis. The inclusion criteria were (i) cases that had undergone complete surgical resection and (ii) cases for which preoperative CT and/or MRI were available.

Five patients with ASCPLTs (three male patients and two female patients; median age, 55 years; age range, 53-87 years) were identified at our institution. Table 1 summarizes the clinical data from five institutional ASCPLT cases, and Tables 2, 3 summarize CT/MRI imaging features from these cases.

**Table 1:** Clinical data from five institutional ASCPLT cases ASCPLT: atypical spindle cell/pleomorphic lipomatous tumor, NC: non-contrast, NA: not available, CE: contrast-enhanced

Case	Age	Sex	Location	Symptom	Operation	CT	MRI	Metastasis	Recurrence	Postoperative follow-up period (M)
1	53	M	Upper arm	No	Total resection	NC	CE	No	No	12
2	55	M	Forearm	No	Total resection	NC	CE	No	No	13
3	55	M	Cheek	No	Total resection	NA	NC	No	No	7
4	77	F	Thigh	No	Total resection	NC	CE	No	No	30
5	87	F	Thigh	Pain	Total resection	CE	CE	No	No	54

**Table 2:** CT/MRI imaging features from five institutional ASCPLT cases ASCPLT: atypical spindle cell/pleomorphic lipomatous tumor, MD: maximum diameter, T1WI: T1-weighted image, SI: signal intensity, T2WI: T2-weighted image, FC: fatty components, NA: not available.

Case	MD (mm)	Depth	Margin	Capsule	T1WI SI	T2WI SI	CT attenuation	FC	Non-FC	SI of non-FC on T2WI	Predominant component
1	145	Superficial	Well-defined	Yes	High, Low	High, Low	Fat, soft-tissue	Yes	Yes	Markedly high	Non-fatty
2	62	Deep	Well-defined	No	Low	High	Fat, soft-tissue	Yes	Yes	Markedly high	Non-fatty
3	39	Superficial	Well-defined	Yes	High, Low	High, Low	NA	Yes	No	Iso	Fatty
4	177	Deep	Well-defined	No	High, Low	High, Low	Fat, soft-tissue	Yes	Yes	Iso	Fatty
5	226	Deep	Well-defined	No	High, Low	High, Low	Fat, soft-tissue	Yes	Yes	Iso	Fatty

**Table 3:** (Continued) CT and MRI imaging features from five institutional ASCPLT cases ASCPLT: atypical spindle cell/pleomorphic lipomatous tumor, SI: signal intensity, FS: fat-suppressed, NA: not available, FC: fatty component, HU: Hounsfield unit, MLS: myxoid liposarcoma, ALT: atypical lipomatous tumor, SCL: spindle cell lipoma.

Case	Peritumoral edema	Septa	High SI on FST2WI	Contrast enhancement	The degree of enhancement	Calcification	CT value of non-FC (HU)	Imaging pattern
1	No	Yes	Solid	Septal/linear	Mild	No	28	MLS
2	No	No	Solid	Nodule	Mild	No	27	MLS
3	No	Yes	No	NA	NA	NA	NA	ALT
4	No	Yes	Hazy	Septal/linear	Mild	No	30	SCL
5	No	Yes	Hazy	Septal/linear	Mild	Yes	27	SCL

Case 1

**Figure 1:**
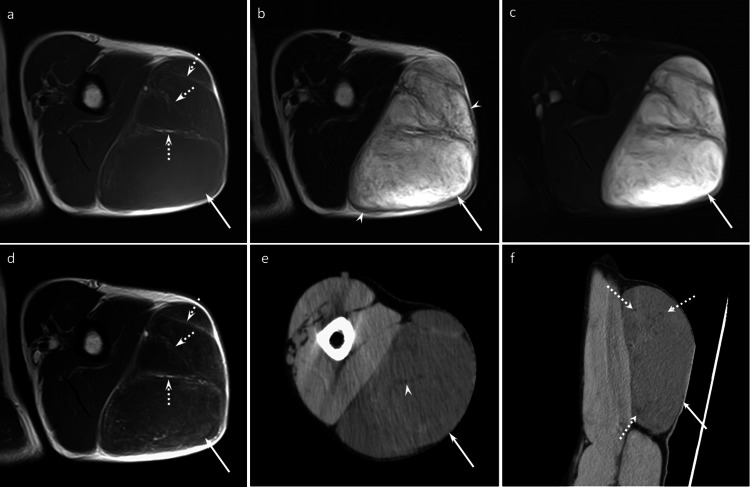
A 53-year-old man with an ASCPLT in the left upper arm Axial T1WI (a), axial T2WI (b), and axial FST2WI (c) show a well-defined subcutaneous non-fatty mass (arrows) with septal/linear fatty components (dotted arrows) and a capsule (arrowheads). The non-fatty components exhibit marked hyperintensity on T2WI. Axial CET1WI (d) shows septal/linear enhancement. Axial CT (e) and coronal CT (f) display a well-defined subcutaneous non-fatty mass (arrow) with a septal/linear fatty area (arrowhead). The imaging findings are consistent with myxoid liposarcoma pattern. T1WI: T1-weighted image, T2WI: T2-weighted image, FS: fat-suppressed, CE: contrast-enhanced.

A 53-year-old man presented with a five-year history of a painless mass in his upper arm. On MRI, a well-defined subcutaneous non-fatty mass with septal/linear fatty components was observed. The non-fatty components exhibit marked hyperintensity on the T2-weighted image (Figure 1). No metastasis was detected in the whole-body scan. The patient underwent complete tumor resection and did not experience recurrence for 12 months. Histologically, the mass was composed of proliferative spindle-shaped cells displaying mild atypia, with an abundant myxoid matrix and fibrous stroma. A small amount of fatty tissue was present within the tumor. Immunohistological examination revealed positivity for CD34 and negativity for MDM2 and DDIT3. The histopathological diagnosis was an ASCPLT.

Case 2

**Figure 2:**
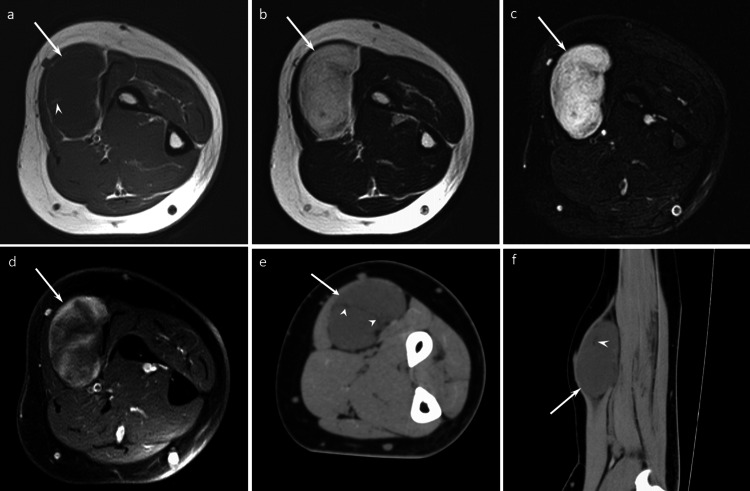
A 55-year-old man with an ASCPLT in the left forearm Axial T1WI (a), axial T2WI (b), and axial FST2WI (c) demonstrate a well-defined intermuscular non-fatty mass (arrows) with septal/linear fatty components (arrowhead) and no capsule. The non-fatty components display marked hyperintensity on T2WI. Axial FSCET1WI (d) shows nodular heterogeneous enhancement. Axial CT (e) and Sagittal CT (f) show a well-defined intermuscular non-fatty mass (arrow) containing a small fatty area (arrowhead). The imaging findings are consistent with myxoid liposarcoma pattern.

A 55-year-old man presented with a one-year history of a painless forearm mass. MRI demonstrated a well-defined intermuscular non-fatty mass with septal/linear fatty components. The non-fatty components display marked hyperintensity on the T2-weighted image (Figure 2). He underwent a body CT, and no metastasis was detected. The patient received total tumor resection and no recurrence was observed for 13 months. The tumor consists of myxoid matrix and fibrous stroma containing spindle cells and adipose components. Immunohistochemical analysis showed positivity for CD34 and negativity for MDM2 and CDK4. Histopathological examination confirmed the diagnosis of an ASCPLT.

Case 3

**Figure 3:**
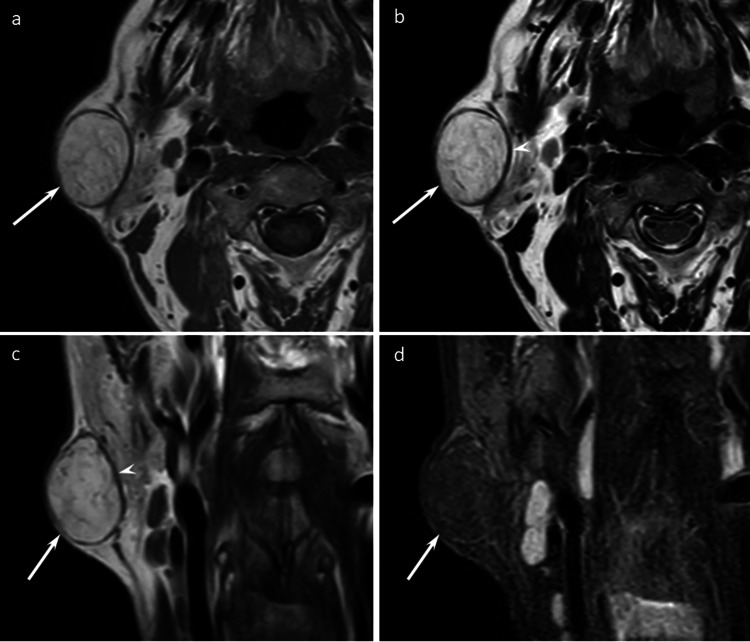
A 55-year-old man with an ASCPLT in the right cheek Axial T1WI (a), axial T2WI (b), coronal T2WI (c), and coronal FST2WI (d) reveal a well-defined subcutaneous fatty mass (arrows) with a clear capsule (arrowheads). The mass shows fat signal intensity without any non-fatty components. The imaging findings are consistent with an atypical lipomatous tumor pattern.

A 55-year-old man presented with a painless mass on his cheek for the past 10 years. MRI revealed a well-defined subcutaneous fatty mass without any non-fatty components (Figure 3). As the tumor was preoperatively suspected to be lipoma, a CT scan was not performed. The tumor was completely removed, and no recurrence was noted during the seven-month follow-up period. Histopathological examination revealed a fatty tumor surrounded by collagen fibers, exhibiting nuclear enlargement and adipocytes of variable size. Immunohistochemical analysis demonstrated CD34 positivity, MDM2 negativity, and loss of RB1 expression. The tumor was histopathologically diagnosed as an ASCPLT.

Case 4

**Figure 4:**
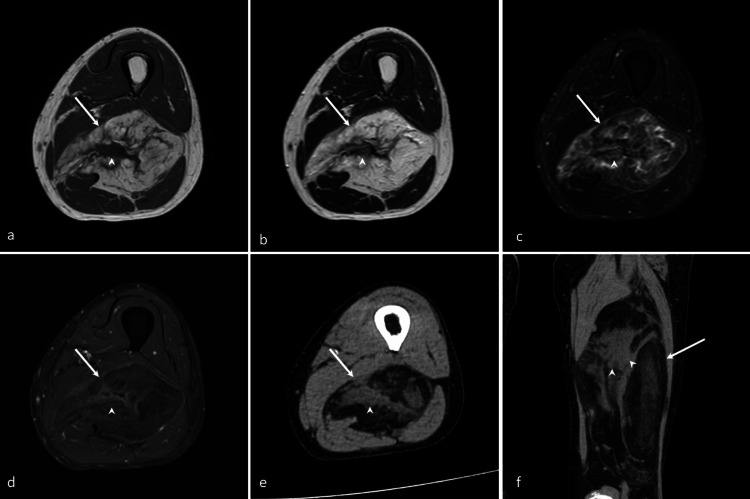
A 77-year-old woman with an ASCPLT in the thigh Axial T1WI (a) and axial T2WI (b) show well-defined intermuscular fatty mass (arrows) containing non-fatty components (arrowhead). Axial FST2WI (c) and axial FSCET1WI (d) demonstrate low signal intensity and mild enhancement of non-fatty components, respectively. Axial CT (e) and coronal CT (f) show a well-defined intermuscular fatty mass (arrow) with non-fatty components (arrowhead). The imaging findings are consistent with a spindle cell lipoma pattern.

A 77-year-old woman presented with a painless mass in her thigh one month ago. MRI showed a well-defined intermuscular fatty mass with central non-fatty components. The non-fatty component was low signal intensity on T2-weighted images and mild enhancement on contrast-enhanced T1-weighted images (Figure 4). There was no metastasis found in his CT scan. Total resection of the tumor was performed, and no recurrence was observed for 30 months. Histopathologically, the tumor consisted predominantly of adipocytic components with abundant myxoid stroma and fibrous tissue. Immunohistochemical staining revealed positivity for CD34, negativity for MDM2, and loss of RB1 expression. The histopathological diagnosis was an ASCPLT.

Case 5

**Figure 5:**
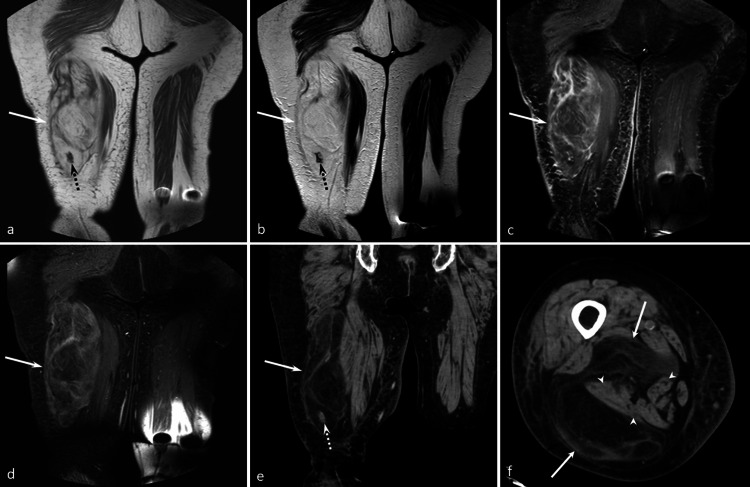
An 87-year-old woman with an ASCPLT in the right thigh Coronal T1WI (a), coronal T2WI (b), coronal FST2WI (c), and coronal FSCET1WI (d) demonstrate a well-defined intermuscular fatty mass (arrows) containing non-fatty components (dotted arrows). Coronal CT (e) shows a well-defined intermuscular fatty mass (arrow) with non-fatty components (dotted arrow). Axial CT (f) shows well-defined intermuscular fatty mass (arrow) with compressed hamstring muscles. The imaging findings are consistent with a spindle cell lipoma pattern.

An 87-year-old woman presented with a painful mass on her thigh that had appeared one week earlier. MRI demonstrated a well-defined intermuscular fatty mass containing non-fatty components (Figure 5). Her CT scans showed no evidence of metastasis. The patient received removal of the entire tumor and had no recurrence for 54 months. The tumor was composed of mature adipose tissue containing variable proportions of mild to moderately atypical spindle cells and lipoblasts. Immunohistochemical staining demonstrated positivity for CD34, but negativity for MDM2 and CDK4. These histopathological findings were consistent with an ASCPLT.

## Discussion

Systematic review

**Figure 6:**
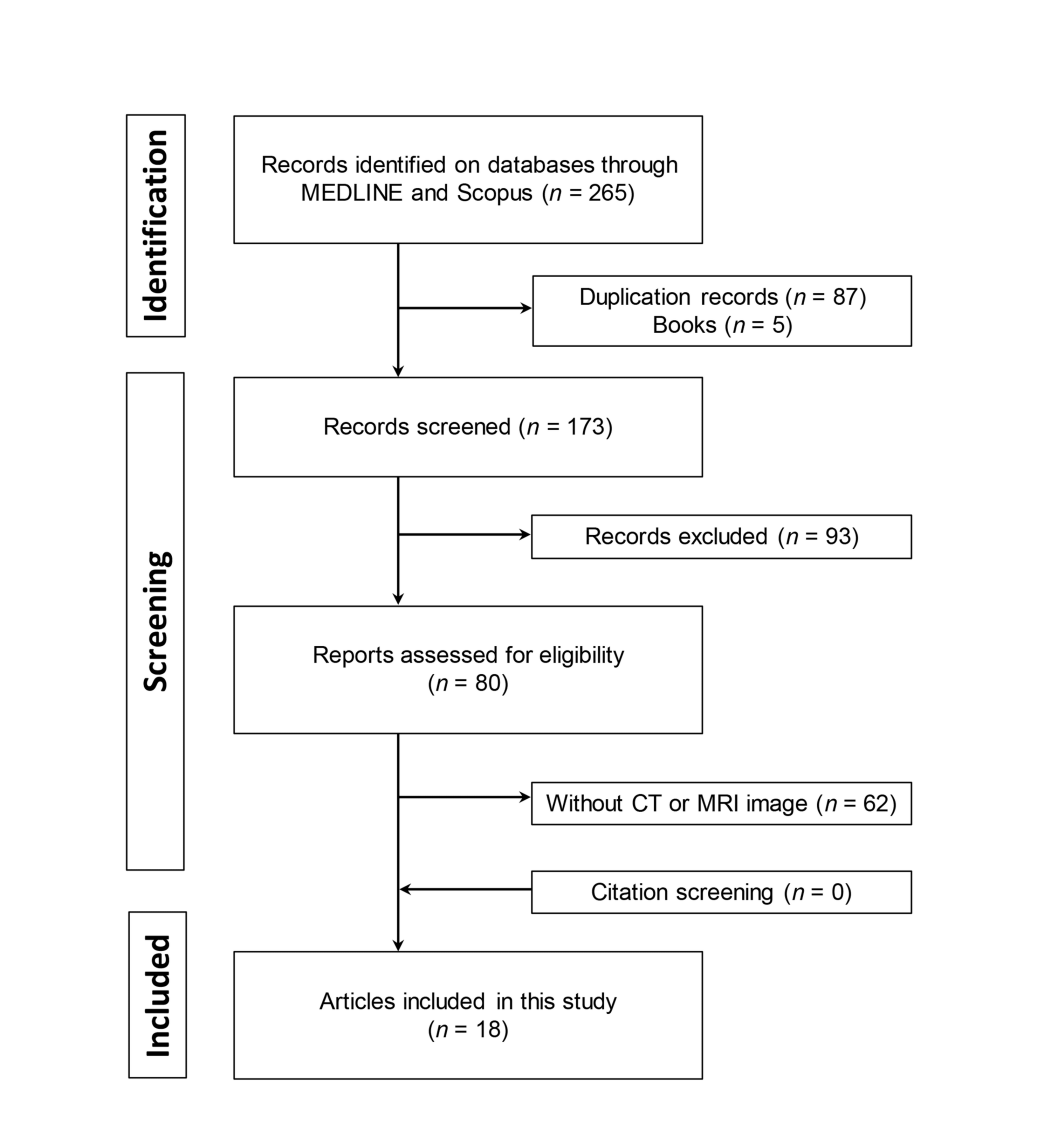
Flowchart illustrating the article selection process based on the PRISMA guidelines PRISMA: Preferred Reporting Items for Systematic Reviews and Meta-Analyses

A literature search was conducted using MEDLINE via PubMed and Scopus, following the PRISMA 2020 guidelines [23]. We searched for publications that included imaging findings of ASCPLTs, covering the period from January 1, 2017, to June 9, 2025. The following keywords were used: (“atypical spindle cell lipomatous tumor”) OR (“atypical pleomorphic lipomatous tumor”) OR (“atypical spindle cell/pleomorphic lipomatous tumor”). Two reviewers screened the titles and abstracts of the articles and assessed the availability of the full texts. The inclusion criteria were (i) cases with histopathologically confirmed ASCPLTs, (ii) availability of preoperative CT, MRI, and/or FDG-PET-CT imaging, and (iii) articles written in English or Japanese. The exclusion criteria were (i) non-peer-reviewed publications, such as books and conference abstracts, and (ii) articles without accessible full text. Additionally, citation tracking of relevant articles was performed. Ultimately, 18 patients from 18 articles were included (Figure 6).

Image Analysis

Two radiologists, with 26 and 12 years of experience in interpreting musculoskeletal imaging, were unblinded to clinical information and jointly reviewed all ASCPLT case images from both our institution and literature.

The reviewers initially assessed qualitative imaging features, including lesion depth (superficial or deep), margin (well-defined or ill-defined), presence or absence of a capsule, signal intensity on T1-weighted images (low, mixed low and high, or high), signal intensity on T2-weighted images (low, intermediate, or high), CT attenuation characteristics (fat, fat and soft tissue, or soft tissue), presence or absence of fatty components, presence or absence of non-fatty components, signal intensity of non-fatty components on T2-weighted images (iso-, mildly high, or markedly high signal intensity), predominant component (fatty or non-fatty ), presence or absence of peritumoral edema, presence or absence of septa, presence or absence of high signal intensity on fat-suppressed T2-weighted images, contrast enhancement (none, septal/linear, or nodular), degree of contrast enhancement on CT and/or MRI (mild or marked), and presence or absence of calcification on CT. Lesions were categorized as superficial if located within the subcutaneous tissue above the fascial plane. A capsule was defined as a hypointense rim on T2-weighted images. Signal intensity on T1-weighted images was categorized as low or high based on similarity to muscle and fat, respectively. For T2-weighted images, low, intermediate, and high signal intensities were defined by resemblance to muscle, lymph nodes, and fat, respectively. Fatty and non-fatty components were identified as nodular regions >1cm on CT, T1-weighted images, or T2- and fat-suppressed T2-weighted images. Peritumoral edema was evaluated on fat-suppressed T2-weighted images. Septa were evaluated within both fatty and non-fatty components on CT, T1-weighted images, or T2-weighted images. On fat-suppressed T2-weighted images, a hazy hyperintense area was defined as ill-defined with a slight degree of hyperintensity, while a solid hyperintense area was defined as well-defined with moderate to marked hyperintensity [24].

Finally, the reviewers categorized the imaging appearances into six different patterns: atypical lipomatous tumor, spindle cell lipoma, myxoid liposarcoma, dedifferentiated liposarcoma, myxofibrosarcoma, or undifferentiated pleomorphic sarcoma. These imaging patterns were evaluated in cases where CT or T2-weighted images were available. Since T2-weighted images alone do not allow reliable evaluation of fat content, only cases with definitive identification of fatty tissue were included in this classification. The imaging pattern definitions were as follows: atypical lipomatous tumor, a fatty lesion without any non-fatty areas; spindle cell lipoma, a lesion predominantly composed of fat, with localized non-fatty components; myxoid liposarcoma, a lesion with mainly non-fatty components that appear as high signal intensity on T2-weighted images or low attenuation on CT; dedifferentiated liposarcoma, a lesion with predominantly non-fatty components showing intermediate signal intensity on T2-weighted images or soft tissue attenuation on CT; myxofibrosarcoma, a non-fatty lesion exhibiting high signal intensity on T2-weighted images or low attenuation on CT, without any fatty components; and undifferentiated pleomorphic sarcoma, a non-fatty lesion with intermediate signal intensity on T2-weighted images or soft tissue attenuation on CT, also lacking fatty components [24-27].

Statistical Analysis

Statistical analyses were performed using IBM SPSS Statistics for Windows, Version 28 (Released 2021; IBM Corp., Armonk, New York, United States). Interobserver variability of qualitative assessments was calculated using the Kappa statistics.

Results

Study Selection

A total of 265 articles from the initial database search, and 92 articles were excluded before the screening. After excluding 155 articles by the screening, the database search yielded 18 articles of 18 patients with ASCPLTs (11 male patients and seven female patients; median age, 60 years; age range, 13-78 years) [5-22]. Table 4 summarizes the clinical data from previous studies. In addition to the five cases identified at our institution, total of 23 patients with ASCPLTs were included in this study (14 male patients and nine female patients; median age, 60 years; age range 13-87 years).

**Table 4:** Clinical data from all ASCPLT cases reported in the literature CE: contrast-enhanced, T1WI: T1-weighted image, T2WI: T2-weighted image, FS: fat-suppressed, NA: not available, STIR: short-tau inversion recovery, NC: non contrast, ASCPLT:  atypical spindle cell/pleomorphic lipomatous tumor

Reference	Year	Age/Sex	Size (mm)	Location	CT	MRI	FDG-PET-CT
Kirisawa et al. [6]	2018	74/M	34	Retroperitoneum	CE	CET1WI	NA
Wehrle et al. [7]	2020	58/M	138	Shoulder	NA	T2WI, FST2WI	NA
Yoshida et al. [8]	2020	74/M	7	Tongue	NA	CET1WI	NA
Sugita et al. [9]	2022	78/F	122	Thigh	NA	T1WI, T2WI, STIR, CET1WI	NA
Bae et al. [10]	2022	18/F	380	Abdominal cavity	CE	NA	NA
Hirsiger et al. [11]	2022	31/F	32	Hand	NA	CET1WI	NA
Bhattarai et al. [12]	2022	60/M	100	Abdominal wall	CE	NA	NA
Ichikawa et al. [13]	2022	64/M	160	Buttock	NA	T1WI, T2WI, STIR, CET1WI	Yes
Graja et al. [14]	2022	77/F	18	Tongue	NA	T2WI	NA
Lugwaja et al. [15]	2023	52/M	359	Retroperitoneum	CE, NC	NA	NA
Ahn et al. [16]	2023	57/M	NA	Orbit	NC	T2WI	NA
Cheng et al. [17]	2023	38/M	115	Psoas muscle	NA	FST2WI, CET1WI	NA
Iseed et al. [18]	2024	13/M	114	Thigh	NA	FST2WI, CET1WI	NA
Batra et al. [19]	2024	20/F	73	Vulva	NA	T1WI, T2WI	NA
Fujibuchi et al. [20]	2024	74/F	NA	Thigh	NA	T1WI, T2WI, CET1WI	Yes
Perret et al. [5]	2024	78/M	55	Chest wall	CE	NA	NA
De Roose et al. [21]	2025	60/F	160	Pelvis	NA	T1WI, FST2WI, CET1WI	NA
Hwang et al. [22]	2025	78/M	NA	Buttock	NA	T1WI, FST2WI	Yes

Clinical Characteristics

Table 5 presents the clinical characteristics of the 23 ASCPLT cases. Tumor locations were as follows: thigh (n = 5), head and neck (n = 4), lower back (n = 2), retroperitoneum (n = 2), buttock (n = 2), and other sites (n = 8). Among 20 patients with symptom data, seven (35%) reported pain and 13 (65%) were asymptomatic. No local recurrence was observed in any of the 14 patients with follow-up data during a median observation period of 12 months.

**Table 5:** The clinical characteristics of 23 ASCPLT cases ASCPLT: atypical spindle cell/pleomorphic lipomatous tumor. Data are the numbers of patients with percentages in parentheses.

Clinical and imaging feature	
Median age (years) [range]	60 [13–87]
Male-to-female ratio	14:9
Location	
Thigh	5/23 (22%)
Head and neck	4/23 (18%)
Lower back	2/23 (9%)
Retroperitoneum	2/23 (9%)
Buttock	2/23 (9%)
Upper arm	1/23 (4%)
Forearm	1/23 (4%)
Hand	1/23 (4%)
Upper back	1/23 (4%)
Chest wall	1/23 (4%)
Abdominal wall	1/23 (4%)
Abdominal cavity	1/23 (4%)
Vulva	1/23 (4%)
Symptom (n = 20)	
Pain	7/20 (35%)
No symptom	13/20 (65%)
Sarcoma component	2/23 (9%)
Recurrence (n = 14)	0/14 (0)
Median follow-up duration (month) [range] (n = 14)	12 [3–54]

Imaging Characteristics

Table 6 presents the imaging features of the 23 ASCPLT cases. The median maximum lesion diameter was 115 mm. Lesion depth was superficial in 14/23 (61%) cases and deep in 9/23 (39%). The tumor margins were well-defined in 20/23 cases (87%) and ill-defined in 3/22 (13%). A capsule was seen on T2-weighted images in 9/12 (75%) cases. On T1-weighted images, low signal intensity was observed in 1/11 (9%) cases, mixed high and low signal intensity in 8/11 (73%), and high signal intensity in 2/11 (18%). On T2-weighted images, low and high signal intensity appeared in 5/12 (42%), intermediate and high in 2/12 (16%), and high signal intensity in 5/12 (42%) (Figures 1-5). CT attenuation showed fat and soft-tissue characteristics in 8/11 (72%) cases and soft-tissue attenuation alone in 3/11 (28%) (Figures 1,2,4,5). Fatty components were observed in 14/18 (78%) cases, and non-fatty components in 15/18 (83%). Among nine cases where signal intensity of non-fatty components was assessed relative to muscle on T2-weighted images, 4/9 (44%) showed isointensity, 1/9 (12%) mild hyperintensity, and 4/9 (44%) marked hyperintensity. Non-fatty components were predominant in 13/18 (72%) cases. Peritumoral edema was seen in 1/11 (9%) cases, while intratumoral septa were present in 12/17 (71%). On fat-suppressed T2-weighted images, hazy hyperintensity was observed in 4/10 (40%) cases and solid hyperintensity in 7/10 (70%). Intratumoral contrast-enhancement was observed in 13/16 (81%) cases, with septal/linear enhancement seen in 8/13 (50%) and nodular enhancement in 9/13 (56%). The degree of contrast enhancement on CT and/or MRI was mild in 7/13 (54%) and marked in 6/13 (46%) cases. Calcification was present in 1/10 (10%) cases. The median maximum standardized uptake value was 2.8 (n = 3).

**Table 6:** The imaging features of 23 ASCPLT cases ASCPLT: atypical spindle cell/pleomorphic lipomatous tumor, SI: signal intensity, T2WI: T2-weighted image, FC: fatty component, FS: fat-suppressed, SUVmax: maximum standardized uptake value. Data are the numbers of patients with percentages in parentheses.

Clinical and imaging feature	n = 23	Kappa value
Median size (mm, n = 20) [range]	115 [7–380]	
Depth		0.73
Superficial	14/23 (61%)	
Deep	9/23 (39%)	
Margins		0.40
Well-defined	20/23 (87%)	
Ill-defined	3/23 (13%)	
Capsule (n = 12)	9/12 (75%)	0.50
SI on T1WI (n = 11)		1.00
Low SI	1/11 (9%)	
High and low SI	8/11 (73%)	
High SI	2/11 (18%)	
SI on T2WI (n = 12)		0.83
Low and high SI	5/12 (42%)	
Intermediate and high SI	2/12 (16%)	
High SI	5/12 (42%)	
CT attenuation (n = 11)		0.23
Fat and soft-tissue attenuation	8/11 (72%)	
Soft-tissue attenuation alone	3/11 (28%)	
FC (n = 18)	14/18 (78%)	0.54
Non-FC (n = 18)	15/18 (83%)	1.00
SI of non-FC on T2WI (n = 9)		1.00
Iso- SI	4/9 (44%)	
Mildly high SI	1/9 (11%)	
Markedly high SI	4/9 (44%)	
Predominant components (n = 18)		0.61
FC	5/18 (28%)	
Non-FC	13/18 (72%)	
Peritumoral edema (n = 11)	1/11 (9%)	0.62
Intratumoral septa (n = 17)	12/17 (71%)	0.75
High signal intensity on FST2WI (n = 11)		
Present	10/11 (91%)	1.00
Hazy (n = 10)	4/10 (40%)	0.79
Solid (n = 10)	7/10 (70%)	0.79
Contrast enhancement on CT and/or MRI (n = 16)		
Present	13/16 (81%)	0.45
Septal/linear (n = 13)	8/13 (62%)	0.62
Nodular (n = 13)	9/13 (69%)	0.80
The degree (n = 13)		0.84
Mild	7/13 (54%)	
Marked	6/13 (46%)	
Calcification (n = 10)	1/10 (10%)	1.00
Median SUVmax (n = 3) [range]	2.8 [2.5–3.48]	

Table 7 shows the frequency of each imaging pattern and summarizes the imaging findings of ASCPLTs. The imaging patterns were classified into five categories: atypical lipomatous tumor (3/17, 18%) (Figure 3), spindle cell lipoma (3/17, 18%) (Figures 4, 5), myxoid liposarcoma (5/17, 30%) (Figures 1, 2), dedifferentiated liposarcoma (4/17, 22%), and undifferentiated pleomorphic sarcoma (2/17, 12%). No cases exhibited a myxofibrosarcoma pattern. The inter-observer agreement was 0.54, as determined using the kappa statistic.

**Table 7:** The frequency of each imaging pattern and summary of imaging findings of ASCPLTs ASCPLT: atypical spindle cell/pleomorphic lipomatous tumor, FC: fatty component. Data are the numbers of patients with percentages in parentheses.

Imaging patterns	
Atypical lipomatous tumor	3/17 (18%)
Spindle cell lipoma	3/17 (18%)
Myxoid liposarcoma	5/17 (30%)
Dedifferentiated liposarcoma	4/17 (22%)
Myxofibrosarcoma	0/17 (0)
Undifferentiated pleomorphic sarcoma	2/17 (12%)
Summary of imaging findings	
Median age	60 years
Median size	115 mm
Depth–Superficial	61%
Capsule	75%
Margins–Well-defined	87%
FC	78%
Non-FC	83%
Predominant components–Non-FC	72%

Discussion

This study is the first to provide a detailed description of the imaging features of ASCPLTs. The characteristic imaging findings included large size (>100 mm), superficial location, well-defined margins, presence of a capsule, and predominantly non-fatty components. ASCPLTs showed a wide range of imaging appearances, which corresponded to varying proportions of fat and non-fatty tissues, as well as different types of non-fatty components.

In this study, ASCPLTs were observed primarily in middle-aged patients. The median age of ASCPLT patients in the literature ranges from 58 to 61 years [28-30], consistent with our findings. Patients with myxoid liposarcoma tended to be younger than those with ASCPLTs [31], whereas those with dedifferentiated liposarcoma, undifferentiated pleomorphic sarcoma, or myxofibrosarcoma were generally older [25,32]. The tendency to occur in middle-aged individuals is a notable clinical feature of ASCPLTs.

The size of ASCPLTs in this study was relatively large. Previous studies describing the pathological features of ASCPLTs reported sizes ranging from 4.0 to 5.4 cm [28,29,33]. The tumors reported in those studies were smaller than those in this study. This is likely due to selection bias, which means that larger cases are more likely to undergo preoperative radiological imaging.

Superficial lesions were more frequent than deep lesions in this study. Earlier pathological research indicated that superficial lesions accounted for 57%-65% of cases [28,33]. Among soft-tissue tumors that resemble ASCPLTs, spindle cell lipomas tend to occur more often in the superficial layer compared to the deep layer [24,34]. Conversely, other soft-tissue tumors such as atypical lipomatous tumors, dedifferentiated liposarcoma, and myxofibrosarcoma more commonly develop in the deep layer [25,31]. Therefore, a superficial location may be a relatively distinctive feature of ASCPLTs.

The present study showed that well-defined margins and capsules were common imaging features, whereas peritumoral edema was rare. Since well-defined margins are frequently seen in other soft-tissue tumors that resemble ASCPLTs, the characteristics of the tumor margin may not be particularly useful in distinguishing ASCPLTs from other tumors. Meanwhile, encapsulated lesions were found in 40% of spindle cell lipoma [34]. Although capsule presence has not been widely evaluated in other soft-tissue tumors, encapsulated lesions are uncommon in malignant soft-tissue tumors due to their infiltrative and aggressive behavior. For this reason, peritumoral edema and the tail sign are often observed in malignant soft-tissue tumors [25]. Therefore, well-demarcated, encapsulated lesions without peritumoral edema are distinguishing features that help separate ASCPLTs from malignant soft-tissue tumors.

This study also found that non-fatty components were predominant in ASCPLTs and the signal intensity of these non-fatty components on T2-weighted images ranged from iso-signal intensity to high signal intensity compared to muscle. Pathologically, ASCPLTs contain varying amounts of pleomorphic spindle cells, collagenous stroma, and/or myxoid extracellular matrix [1,28]. The imaging appearances of the non-fatty components reflect this histological diversity. On T2-weighted images, iso-signal intensity corresponds to collagenous stroma, mildly high signal intensity corresponds to hypercellular areas or solid growth, and markedly high signal intensity corresponds to myxoid stroma [28]. Because ASCPLTs include diverse histological elements, the signal intensity of their non-fatty components on T2-weighted images can vary widely.

ASCPLTs exhibited a variety of imaging patterns. Accurate imaging diagnosis of ASCPLTs is difficult because of the wide range of imaging features. Similarly, ASCPLTs have a broad histological differential diagnosis including atypical lipomatous tumor/well-differentiated liposarcoma, dedifferentiated liposarcoma, and pleomorphic liposarcoma [28]. Consequently, making a pathological diagnosis of ASCPLTs can also be challenging when relying on smaller tissue samples [2]. If radiologists mention the possibility of ASCPLTs based on imaging findings, a detailed pathological examination including immunohistochemistry intra-operative frozen section diagnosis can be performed. Radiologists should be well-informed about ASCPLTs and consider them when encountering a subcutaneous fatty mass with a predominant non-fatty component and a capsule in middle-aged adults.

The management approach for ASCPLTs has not been standardized. ASCPLTs are benign adipocytic tumors with a low risk of local recurrence and no documented risk of metastasis, despite the presence of sarcoma components in rare cases [2]. The most commonly performed surgical treatment is complete excision with clear margins [2]. Wide surgical margins are not necessary for the resection of ASCPLTs. Therefore, if preoperative imaging enables an accurate diagnosis of ASCPLTs, a limited resection margin may be applied. In addition, conservative observation may be considered for patients who are poor candidates for surgery. Although preoperative imaging diagnosis of ASCPLTs may have a practical impact on management, further studies are needed to clarify the imaging characteristics of ASCPLTs.

The study has several limitations. First, the number of cases from our institution was small due to the rarity of the disease. Second, because the available radiological images from the literature were limited, the imaging findings could not be fully evaluated. In particular, the number of patients who had CT scans (10/23, 43%) and FDG-PET/CT (3/23, 13%) was small. Third, imaging scanners and parameters varied widely and could not be standardized because this study was based on a collection of case reports. Fourth, our review consisted solely of case reports, which may have introduced publication and selection biases. It should be noted that publication bias is likely, particularly for case reports. Cases with large or conspicuous lesions are particularly more likely to be submitted and accepted for publication, potentially biasing the frequency and characteristics of findings. Furthermore, heterogeneity in imaging protocols across institutions, including differences in modality, acquisition parameters, and interpretation criteria, may affect reported rates and limit study comparability. Fifth, since ASCPLTs display a wide range of imaging features, we were unable to identify definitive findings to distinguish ASCPLTs from other soft-tissue tumors.

## Conclusions

This study described the imaging features of ASCPLTs. Large size (>100 mm), superficial location, well-defined margins, presence of a capsule, and predominantly non-fatty components were notable imaging features of ASCPLTs. A definitive diagnosis should be established pathologically because the radiological differential diagnosis of ASCPLTs is broad, and the imaging features overlap with other soft tissue sarcomas. However, if ASCPLTs can be diagnosed or at least suspected preoperatively based on imaging findings, clinicians may be able to avoid overtreatment and misclassification as sarcoma. The key point for radiologists is to consider ASCPLTs when encountering a superficial, well-defined, fat-containing mass with non-fatty components, particularly in a middle-aged adult.
